# Melioidosis parotitis in children

**DOI:** 10.1186/s40409-016-0088-1

**Published:** 2016-12-05

**Authors:** David Dance

**Affiliations:** 1 Senior Clinical Research Fellow/Consultant Microbiologist, Lao-Oxford-Mahosot Hospital-Wellcome Trust Research Unit (LOMWRU), Department of Microbiology, Mahosot Hospital, Vientiane, Lao PDR; 2 Centre for Tropical Medicine & Global Health, Nuffield Department of Clinical Medicine, University of Oxford, Oxford, England UK; 3 Honorary Professor, Faculty of Infectious and Tropical Diseases, London School of Hygiene and Tropical Medicine, London, England UK

**Keywords:** Melioidosis, Parotitis, *B. pseudomallei*, Paediatric, Children

## Abstract

A recent paper published in *JVATiTD* reporting a child in Hainan with parotitis caused by *Burkholderia pseudomallei* misleadingly described parotitis as a rare manifestation of melioidosis. In fact, it is one of the commonest forms of paediatric melioidosis seen in other parts of Southeast Asia, although interestingly not in Australia.

To: The Editor, *Journal of Venomous Animals and Toxins including Tropical Diseases*

Sir,

Melioidosis (*Burkholderia pseudomallei* infection) is an important public health problem in some parts of the world. The incidence has probably been grossly underestimated in the past [1], so it is encouraging that reports have recently begun to emerge from several countries across the tropics, including Hainan island in China [2–4]. The most recent such report, published in your journal, related to a case of parotitis in a child [5]. Unfortunately, this report contained a highly misleading statement about melioidosis parotitis, namely that it was rare and had only been reported following systemic melioidosis. In fact it has been known for many years that parotitis is one of the commonest manifestations of melioidosis in children in SE Asia [6, 7], although interestingly not in Australia [8]. This should have been revealed by a simple literature search using the terms ‘melioidosis’ and ‘parotitis’ – a PubMed search using these terms on 14th November 2016 yielded 16 ‘hits’ including the references cited above.

Whilst I am sure that this was a genuine mistake, I think it is important that this misleading impression is corrected.

Yours sincerely

Prof. David Dance, MB, ChB, MSc, FRCPath
